# Delirium care in crisis: a retrospective analysis of reduced hospitalizations during the COVID-19 pandemic in Germany

**DOI:** 10.1186/s12877-026-07467-x

**Published:** 2026-06-24

**Authors:** Nils Diogo Nellessen, Mohamad Samehni, Sven Hohenstein, Andreas Bollmann, Julius Dengler, Frederick Palm, Juraj Kukolja

**Affiliations:** 1 Faculty of Health, Witten/Herdecke University, Witten, Germany; 2 Department of Neurology and Clinical Neurophysiology, Helios University Hospital Wuppertal, Wuppertal, Germany; 3 Hospital for Psychiatry, Evangelische Stiftung Tannenhof, Remscheid, Germany; 4 Clinical Trial Management & Real World Data, Helios Health Institute, Leipzig, Germany; 5 Faculty of Health Sciences Brandenburg, Brandenburg Medical School, Theodor Fontane, Campus Bad Saarow, Bad Saarow, Germany; 6 Department of Neurosurgery, Helios Hospital Bad Saarow, Bad Saarow, Germany; 7 Department of Neurology, Helios Hospital Schleswig, Schleswig, Germany

**Keywords:** Delirium, COVID-19, Pandemic, Availability of health services, Inpatients, Outpatients, Clinical epidemiology, Geriatrics, Diagnosis-related groups

## Abstract

**Background:**

The COVID-19 pandemic has caused profound shifts in healthcare utilization. Little is known about how this crisis affected the vulnerable group of patients with delirium on a system level. The goals of this study where to examine whether this crisis: (1) altered the number of inpatient admissions for delirium, (2) altered the number of hospital-based outpatient admissions for delirium, (3) affected disease burden, all-cause in-hospital mortality or length of stay for inpatient delirium cases.

**Materials and methods:**

We retrospectively analysed routine and health claim data from 7,720 admissions for delirium from 86 German hospitals and compared pandemic and pre-pandemic data. We included all cases with the main discharge diagnosis from the International Classification of Disease-10 (ICD-10): “F05 Delirium, not induced by alcohol and other psychoactive substances” from 2019 to 2022. The mean age was 80.9 years in the pandemic period and 80.7 years in the pre-pandemic period. Admission rates, length of stay, disease burden (case mix, comorbidities) and complications (i.e., intensive care admission, all-cause in-hospital mortality) were assessed via (generalized) mixed models.

**Results:**

We observed a 20% decrease in inpatient admissions for delirium during the pandemic (*p* < 0.001). This reduction was not offset by hospital-based outpatient care. Case mix index values, individual secondary diagnoses and complications were unchanged or reduced during specific pandemic periods. Moreover, time all-cause in-hospital mortality remained unchanged. The proportion of elective admissions decreased from 18% to 12% (*p* < 0.001). The Length of hospital stay remained unchanged.

**Conclusions:**

The COVID-19 pandemic was associated with a marked reduction in inpatient admissions for delirium, which was not offset by hospital-based outpatient care. Changes in admission patterns and disease burden indicators suggest altered thresholds and pathways for admissions for delirium during the pandemic. These findings point to potential gaps in access to inpatient delirium care during the pandemic. Such real-world evidence is essential for improving healthcare for vulnerable groups, such as those with delirium.

## Background

The COVID-19 (“corona virus disease 2019”) pandemic was an extraordinary event exerting profound pressure on healthcare systems globally. It led to remarkable shifts in how medical services were delivered and utilized. The main changes in German hospitals were widespread suspensions of elective admissions and surgeries, redistribution of wards and triage [1]. Studies on these effects and adaptations can teach us about the inner workings, weaknesses and strengths of our healthcare systems. The consequent impact of altered medical service delivery is unique to each disease because of their specific care pathways, needs, and vulnerabilities. Delirium is prevalent in up to 50% of hospitalised patients [2] and is characterised by sudden onset, fluctuating cognitive deficits [3]. There are multiple causes of delirium, including dementia, hospitalization, surgery, cancer and medical conditions such as infection [4]. Delirium is especially prevalent among elderly and geriatric patients [5], who are also at high risk for severe COVID-19. Delirium not only complicates the clinical course but also has been associated with increased morbidity, mortality and prolonged hospital stays [2]. During the pandemic, delirium gained additional significance as it emerged as both a direct symptom of COVID-19 [6–9] and a complication related to hospitalization itself [10].

The literature on real-world data concerning hospital utilization (in Germany) by cases of patients with delirium is sparse. Several medical specialties experienced a decrease in inpatient admissions [11]. Our previous analysis revealed a decrease in hospital admissions of up to −18% for all neurological conditions (including delirium cases) between 2020 and 2022 compared with 2019, whereas the length of stay was shorter and morbidity indices and all-cause mortality were higher during the pandemic than in the pre-pandemic periods [12]. There is sparse data on the impact of the COVID-19 pandemic on inpatient or outpatient admission for delirium, and the potential changes in disease burden indicators, all-cause mortality, or length of hospital stay.

Therefore, we address the following research questions:

1. Did the COVID-19 pandemic impact the number of inpatient admissions for delirium compared with pre-pandemic times?
2. How did the number of hospital-based outpatient admissions for delirium change due to the COVID-19 pandemic, and how did this change compare to the change in number of inpatient admissions?
3. Were there other changes in healthcare delivery for inpatient admissions for delirium – specifically regarding disease burden indicators, all-cause in-hospital mortality, or length of stay?

## Materials and methods

### Helios hospital structure

This study analysed anonymized retrospective data from routine claims of 86 hospitals in Germany from the Helios hospital group. Individual structural information on the participating hospitals (e.g., size, location, level of care) were not available due to data protection regulations. The Helios hospital network encompasses all care levels from primary care to maximum care, from non-academic to university hospitals all over Germany.

### Data extraction

All the original data had been collected for the purpose of providing clinical care and billing services to health insurance companies. The analysed cohort was part of a larger study cohort in a previous publication [12]. Data were extracted *via* QlikView (QlikTech, Radnor, Pennsylvania, USA).

### Definition of time periods and control periods

The total study period was March 1 st 2020, through December 31 st 2022 compared to the control period January 1 st 2019, through December 31 st 2019. Although the comparison of the pandemic periods to the single year of 2019 does not account for year-to-year fluctuations, this comparison ensures a comparison with the most recent pre-pandemic year. The inclusion of additional years as references could introduce variability related to healthcare policy changes, population demographics or other external factors. The diagnosis-related group (DRG) coding framework in Germany remained largely stable between 2019 and 2022. Pandemic-related documentation requirements and the introduction of additional diagnostic and procedural codes (chapters Z and U) increased coding intensity and thereby the number of recorded secondary diagnoses. These changes did not affect the coding framework for main diagnoses, including ICD-10 chapter F diagnoses such as delirium.

The data were segmented into different virus predominance periods as previously described [4]:

1. Wildtype: March 1 st 2020 – March 7th 2021 (372 days).
2. Alpha: March 8th 2021 – June 25th 2021 (110 days).
3. Delta: June 26th 2021 – January 2nd 2022 (191 days).
4. Omicron: January 3rd 2022 – December 31 st 2022 (362 days).

The different virus predominance periods were compared with the corresponding control periods of 2019 on the same dates. For instance, the alpha predominance period from March 8th 2021, to June 25th 2021, was compared to that from March 8th 2019, through June 25th 2019. As the study period for the wildtype predominance period was more than 365 days, seven days from 2019 were included twice (March 2nd 2019, through March 8th 2019). These control intervals were constructed independently and partly overlapped by design.

### Diagnostic coding system

Diagnoses were made according to the International Statistical Classification of Diseases and Related Health Problems, 10th Revision, German Modification (ICD-10-GM) with the main diagnosis being the primary reason for hospital stay. Secondary diagnoses existed at the time of hospital stay or occurred during the hospital stay and indicated a complicated treatment.

### Inclusion criteria

This study was designed to investigate admissions primarily attributed to delirium. They were operationalized as cases with the main discharge diagnosis coded as “F05: Delirium, not induced by alcohol and other psychoactive substances”. In clinical practice the main discharge diagnosis is chosen through a retrospective assessment of clinicians at the time of hospital discharge. Therefore, the underlying medical condition was not necessarily recognized as delirium before or at the time of admission. This approach captures explicit hospital care pathways in which delirium itself drove hospitalization and resource utilization. Other cases defined by codes of delirium as substance-induced (F10.4: Delirium tremens), or delirium as a secondary diagnosis or via nonspecific symptom codes (i.e., R41.0: Disorientation, unspecified) were intentionally excluded, as these cases are associated with different clinical trajectories and admission pathways. Including such codes would have increased heterogeneity and introduced misclassification bias. This is particularly true in a multicentre analysis. Accordingly, the present study focuses on hospital-based treatment pathways for explicitly recognized delirium and does not aim to capture unrecognized or secondary delirium occurring in the context of other primary diagnoses.

### Demographic information

We extracted age in years and sex data (male or female) to characterize cohorts.

### Definition of inpatient and outpatient cases

“Inpatient” cases received full inpatient hospital care. “Outpatient” cases in this study refer to hospital-based day-care treatments documented within the same administrative and billing system as inpatient cases. Only services coded and reimbursed through the hospital sector were included. Primary care or community-based outpatient treatments were not captured, as they fall under a separate reimbursement framework in the German healthcare system. Hospital-based day-care treatment is primarily intended for post-acute rehabilitation and is not structurally equipped to manage acute diseases.

### Mode of admission

We extracted the mode of admission, meaning that “elective” was a scheduled admission and “urgent” was either due to an unscheduled emergency or an in- or inter-hospital transfer. Elective admissions coded with delirium as the main discharge diagnosis typically reflect planned diagnostic evaluation, multimodal treatment or admission for recurrent or persistent delirium symptoms rather than acute emergencies. For example, a patient may be referred by a general practitioner and scheduled for admission on a later date, presenting directly to the ward (“elective”) rather than being admitted immediately (“urgent”).

### Length of stay

We extracted the length of stay, which was defined by the number of nights in treatment.

### Factors and variables for comorbidities and complications

All variables for comorbidity and complications were analysed for the inpatient cohort only, since they were not available for outpatient cases because of the nature of German DRG data.

The following list contains all variables concerning comorbidities and complications and their operationalization (where applicable):

- Case mix values were calculated and provided by each hospital for inpatient cases as part of routine DRG-based billing data; weights were standardized according to national reimbursement regulations and reflected the relative expected resource use of each hospital case based on diagnoses, procedures, and other factors.
- The Elixhauser comorbidity index (ECI), a widely used and validated measure of overall comorbidity burden based on administrative data, was used to assess the severity of comorbidities [13, 14]. The weighted ECI was calculated using the AHRQ algorithm [15, 16]. Furthermore, the frequency and proportion of each secondary diagnosis contained in the ECI were extracted.
- Total number of coded secondary diagnoses.
- Mechanical ventilation (through codes of operations and procedures (OPS): OPS 8-70x, 8-71x, or duration of ventilation > 0).
- Intensive care admission (through OPS codes 8–980, 8-98d, 8-98f, or duration of intensive care stay > 0).
- All-cause in-hospital mortality (yes or no).

Furthermore, we analysed the occurrence of specific secondary diagnoses selected because of their clinical relevance as typical complications in geriatric patients and mentioning in scientific literature [17, 18]: N17 Acute kidney failure, E86 Volume depletion (exsiccosis), N30 Cystitis, I26 Pulmonary embolism, J09-J18 Influenza and pneumonia, J69.0 Aspiration pneumonia, I80 Thrombosis, phlebitis, and thrombophlebitis.

### Statistical analyses

#### General remarks

Inferential statistics were based on generalized linear mixed models (GLMMs), specifying hospitals as random factors [19]. The effects were estimated using the lme4 package (version 1.1–26) in an R environment for statistical computing (version 4.0.2, 64-bit build) [20, 21]. In all mixed models, we specified varying intercepts for the random factor.

#### Level of significance

For all tests, we applied a two-tailed 5% error criterion for significance. Owing to the exploratory approach of this study, we considered an uncorrected p value of < 0.05 as statistically significant. For linear mixed models, we computed p values based on the Satterthwaite Approximation for the degrees of freedom.

#### Cohort description

To describe the patient characteristics of the cohorts and comorbidities, we employed χ2-tests for categorical variables (sex) and a two-sample t-test for numerical variables (age). We reported the proportions, means, ranges (for age), standard deviations, and p values.

#### Admission patterns

Admission rates were modelled using negative binomial GLMMs with a log link function. We reported daily admissions, incidence rate ratios (IRRs), 95% confidence intervals (CI) and p values.

The admission mode was modelled using logistic GLMMs to compare the proportions of urgent versus elective admissions between time periods. We reported proportions and p values.

**Fig. 1:**
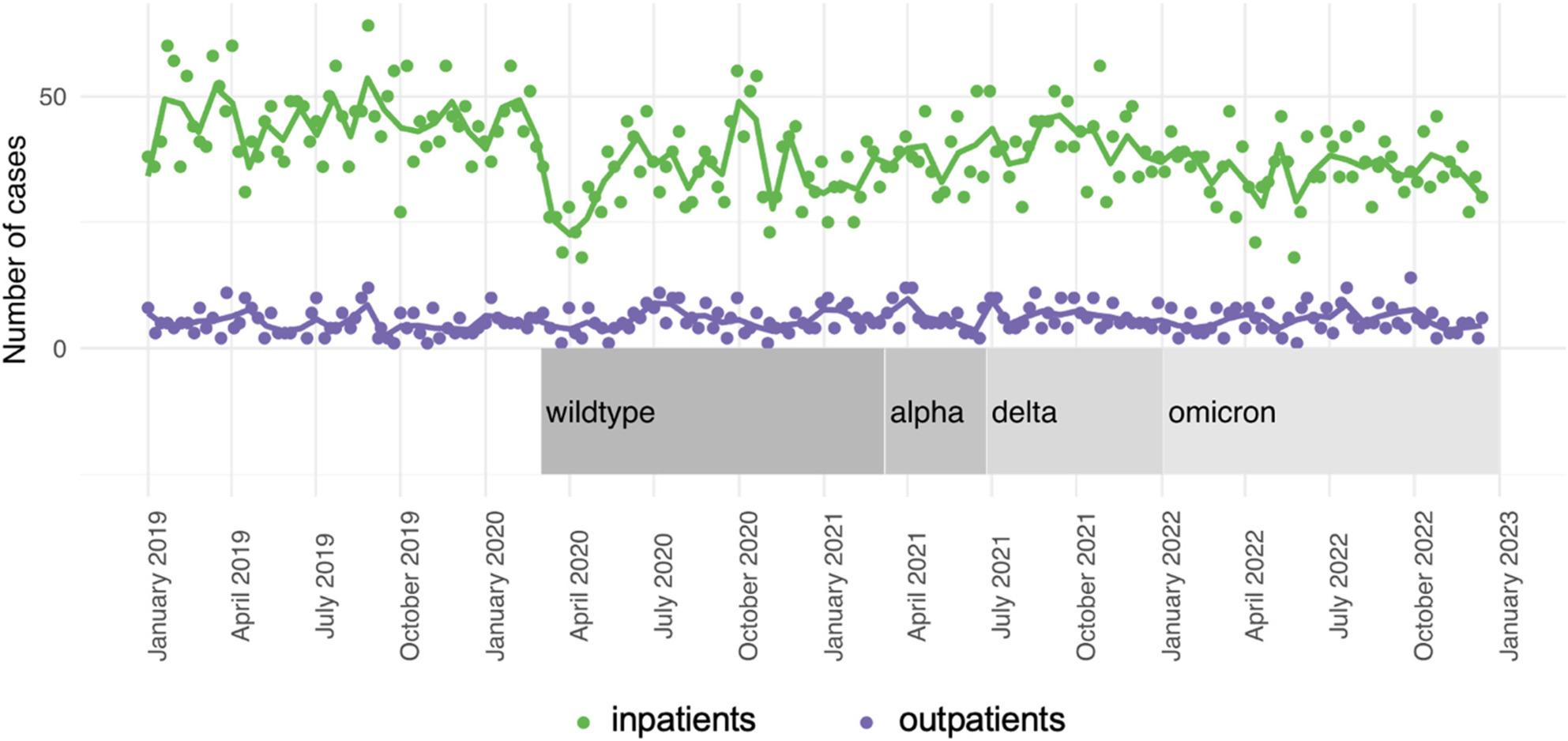
Changes in number of cases for patients with delirium during the COVID-19 pandemic

Length of stay was modelled using linear mixed models. Because of a positively skewed distribution, we applied an inverse hyperbolic sine transformation. We reported means, standard deviations, and p values. For visualization of temporal admission trends in Fig. 1, locally estimated scatterplot smoothing (LOESS) curves were applied to the quarterly admission counts of inpatient and hospital-based outpatient cases.

#### Outpatient cases

Outpatient admission counts were analysed via the same negative binomial GLMM approach as the inpatient rates. No multivariable modelling was applied to outpatient morbidity or mortality because of a lack of comparable data.

#### Cumulative hospitalization deficit

The cumulative hospitalization deficit was computed as the difference between the expected and observed cumulative admission numbers in the study period, expressed as the IRR of the cumulative expected number. The expected number of admissions was defined as the daily average during the 2019 control period.

#### Comorbidities and complications (inpatient cases)

Case mix, ECI, and number of secondary diagnoses were modelled using linear mixed models. The presence of selected complications and individual ECI items were modelled using logistic GLMMs. We reported means, standard deviations and p values. Because the variables Case mix and number of secondary diagnoses were positively skewed, we transformed them using an inverse hyperbolic sine to approximate normal distributions.

Mechanical ventilation, intensive care admission, and in-hospital all-cause mortality were analysed as binary outcomes using logistic GLMMs with hospital as a random effect. We reported proportions and p values.

### Declaration of generative AI and AI-assisted technologies in the writing process

During the preparation of this work, the authors used ChatGPT-4 and − 5.2 from OpenAI (chat.openai.com) to improve the writing of the manuscript, specifically suggesting translations from German to English and offering summaries or restructured passages of our drafts. Springer Nature’s AI tool Curie was used for minor language editing of the final draft. After using these tools, the authors reviewed and edited the content as needed and take full responsibility for the content of the published article.

### Ethics approval and consent to participate

This study was approved by the Ethics Committee of the Medical Faculty of Leipzig University (#490/20-ek). Owing to the retrospective nature of the data and anonymized data extraction, informed consent was not obtained. Helios Health and Helios Hospitals have strict rules regarding data-sharing and detailed restrictions to preserve data privacy. This study was conducted according to the Declaration of Helsinki in its most recent form [22]. Clinical trial number: not applicable.

## Results

### Case numbers and demographics

Case numbers, demographics and mode of admission are detailed in Table 1. The overall number of included inpatient cases was 7,720, of which 5,362 cases were from the study period and 2,358 cases from the control period. The mean age of the inpatients was 80.9 years (SD 9.5; range 13–103) during the study period and 80.7 years (SD 9.7; range 16–102) during the control period (no difference in mean values, *p* = 0.442). Patients from inpatient cases were significantly older in the delta period compared to the control period (*p* = 0.045). The proportion of male inpatients was 47% in total in both, the study and control periods (*p* = 0.818).

**Table 1:** Patient characteristics and case severity for cases with inpatient treatment

Characteristic		**Total**		**Wildtype**		**Alpha**		**Delta**		**Omicron**	
		value	*p*	value	*p*	value	*p*	value	*p*	value	*p*
*Case number*,* N*	*Study*	*5362*		*1840*		*602*		*1108*		*1812*	
	*Control*	*2358*		*2396*		*706*		*1240*		*2341*	
Age, years; mean ± SD	Study	80.9 ± 9.5	0.442	80.9 ± 9.4	0.676	80.3 ± 10.1	0.309	**81.2 ± 9.3**	**0.0455**	81.0 ± 9.5	0.414
	Control	80.7 ± 9.7		80.8 ± 9.7		80.8 ± 9.0		**80.4 ± 10.3**		80.7 ± 9.8	
Male sex, N (proportion)	Study	2505 (47%)	0.818	841 (46%)	0.392	289 (48%)	0.885	523 (47%)	0.961	852 (47%)	0.922
	Control	1109 (47%)		1128 (47%)		335 (47%)		583 (47%)		1096 (47%)	
Urgent admissions, N (proportion)	Study	**4696 (88%)**	**< 0.001**	**1597 (87%)**	**< 0.001**	**535 (89%)**	**< 0.001**	**968 (87%)**	**0.019**	**1596 (88%)**	**< 0.001**
	Control	**1932 (82%)**		**1958 (82%)**		**572 (81%)**		**1040 (84%)**		**1919 (82%)**	
Elixhauser comorbidity score, mean ± SD	Study	9.5 ± 9.8	0.393	10.2 ± 10.0	0.096	9.4 ± 9.8	0.413	9.5 ± 10.2	0.769	**8.7 ± 9.4**	**0.002**
	Control	9.7 ± 9.8		9.7 ± 9.8		9.9 ± 9.9		9.3 ± 9.7		**9.7 ± 9.8**	
Case Mix^T^, mean ± SD	Study	**0.91 ± 0.49**	**0.005**	0.9 ± 0.5	0.486	**0.9 ± 0.7**	**0.010**	0.9 ± 0.4	0.112	0.9 ± 0.4	0.153
	Control	**0.95 ± 0.86**		1.0 ± 0.9		**1.0 ± 0.7**		0.9 ± 1.1		1.0 ± 0.9	
Secondary diagnoses^T^, mean ± SD	Study	**13.7 ± 6.6**	**< 0.001**	**13.6 ± 7.0**	**< 0.001**	14.1 ± 6.4	**< 0.001**	**13.7 ± 6.4**	**< 0.001**	**13.7 ± 6.4**	**< 0.001**
	Control	**11.9 ± 6.2**		**11.9 ± 6.2**		12.0 ± 6.3		**11.6 ± 6.1**		**11.9 ± 6.2**	

^T^: Statistical testing was performed after the data had been transformed via inverse hyperbolic sine to roughly approximate normal distributions; descriptive values are reported as the raw means ± SD. Bold print: significant difference between the study and control periods (*p* < 0.05)

The overall number of included outpatient cases was 1,110, of which 858 were from the study period and 252 from the control period. The mean age in outpatient cases was 78.7 years (SD 13.5; range 4–100) in the study period and 77.6 years (SD 14.9; range 17–100) in the control period (no difference of mean values; *p* = 0.280). The proportion of male outpatients was 46% in the study period and 41% in the control period (no group difference; *p* = 0.206).

### Admission patterns

#### Inpatient admission patterns

**Fig. 2:**
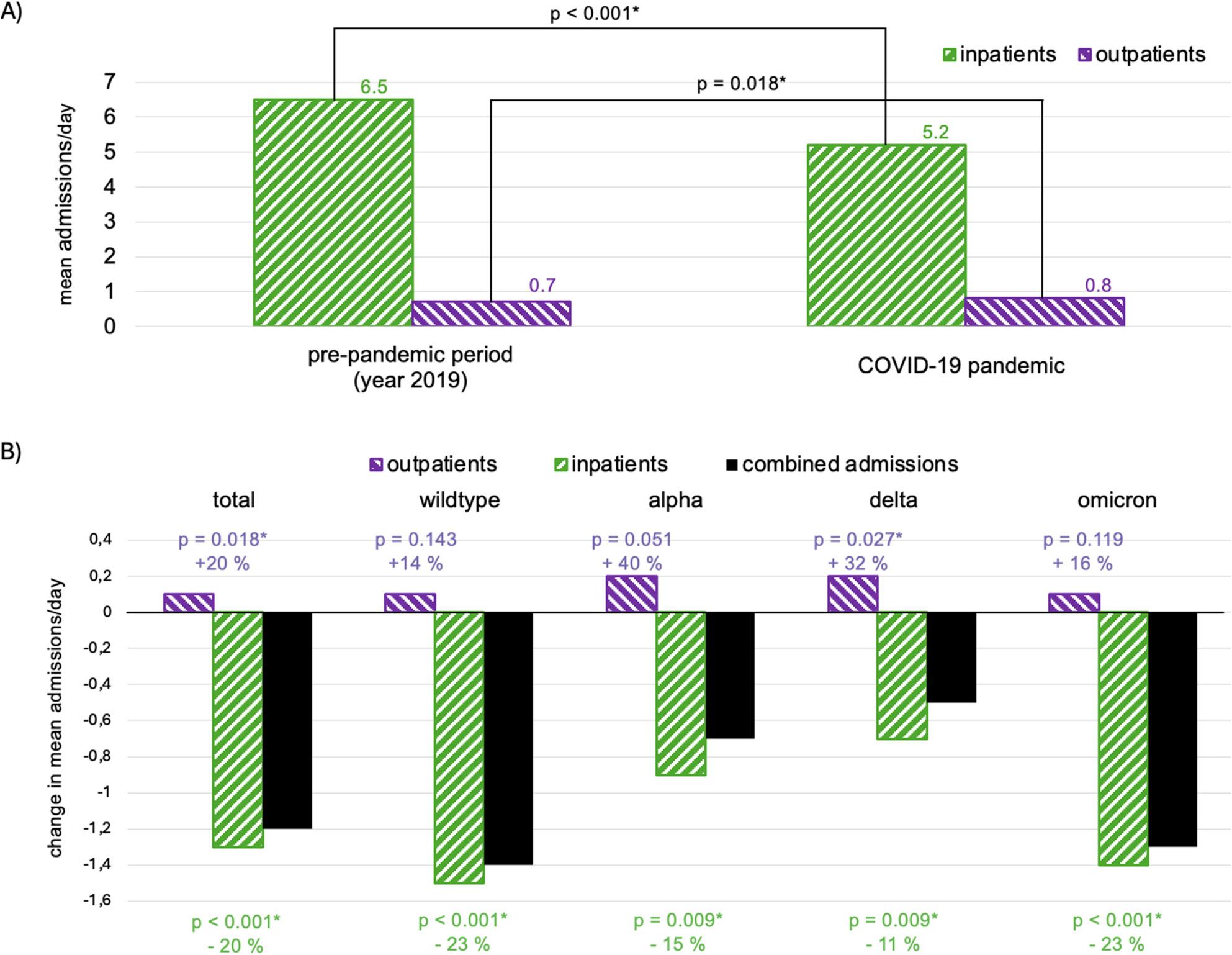
Shift during pandemic: deficit in inpatient admissions, while outpatient admissions barely increased. **A** Mean number of inpatient and hospital-based outpatient admissions per day for cases with delirium as the main discharge diagnosis during the pre-pandemic period (2019) and the COVID-19 pandemic period. **B** Change in mean admissions per day relative to the corresponding control periods, shown for the total period and for pandemic phases with different predominant SARS-CoV-2 variants (wildtype, alpha, delta, omicron). Purple bars indicate hospital-based outpatient admissions, green bars indicate inpatient admissions, and black bars represent the combined change (inpatients + outpatients). * significant results with *p* < 0.05

The data are visualized in Figs. 1 and 2. During the total pandemic period, the mean number of inpatient admissions per day with a main diagnosis of delirium was 5.2 admissions/day (SD 2.6) in the study period compared with 6.5 admissions/day (SD 3.1) in the control period, resulting in an IRR of 0.80 (95% CI 0.76–0.85, *p* < 0.001).

During the wildtype period, the mean number of inpatient admissions per day with a main diagnosis of delirium was 4.9 admissions/day (SD 2.6) in the study period compared with 6.4 admissions/day (SD 3.1) in the control period, resulting in an IRR of 0.77 (95% CI 0.71–0.83, *p* < 0.001).

During the alpha period, the mean number of inpatient admissions per day with a main diagnosis of delirium was 5.5 admissions/day (SD 2.7) in the study period compared with 6.4 admissions/day (SD 2.8) in the control period, resulting in an IRR of 0.85 (95% CI 0.75–0.96, *p* = 0.011).

During the delta period, the mean number of inpatient admissions per day with a main diagnosis of delirium was 5.8 admissions/day (SD 2.9) in the study period compared with 6.5 admissions/day (SD 3.2) in the control period, resulting in an IRR of 0.89 (95% CI 0.81–0.99, *p* = 0.027).

During the omicron period, the mean number of inpatient admissions per day with a main diagnosis of delirium was 5.0 admissions/day (SD 2.4) in the study period compared with 6.3 admissions/day (SD 3.1) in the control period, resulting in an IRR of 0.77 (95% CI 0.72–0.83, *p* < 0.001).

#### Outpatient admission patterns

The mean number of outpatient admissions per day with delirium as the main diagnosis was 0.8 admissions/day (SD 1.0) during the total pandemic period compared with 0.7 admissions/day (SD 0.9) for the control period, resulting in an IRR of 1.20 (95% CI 1.03–1.40, *p* = 0.018).

A statistically significant increase in outpatient admissions per day with delirium as the main diagnosis was also observed during the delta variant predominance period, with a mean of 0.9 admissions/day (SD 1.0) in the study period compared with 0.7 outpatient admissions/day (SD 0.9) in the control period, resulting in an IRR of 1.32 (95% CI 1.03–1.69, *p* = 0.027).

#### Compensatory analyses: relationship of inpatient versus outpatient admissions

Figure 1 displays these data together with LOESS curves illustrating the overall quarterly admission numbers and higher temporal resolution. The largest visual spike in the reduction in the number of inpatient case admissions was in April 2020, during the first wave of COVID-19, with wildtype predominance. The subsequent reductions of case admissions were less pronounced and formed a lower plateau than they did in the pre-pandemic period. Inpatient admission rates remained significantly lower throughout the pandemic than before the pandemic. The statistically significant increases in outpatient cases during the pandemic are numerically much lower than the statistically significant reductions in inpatient cases (cf. Figures 1 and 2). The “sum” values shown in Fig. 2B represent the combined mean number of inpatient and hospital-based outpatient admissions per day for the respective periods.

#### Admission mode (for inpatient cases)

Compared with that in the control periods, the proportion of urgent admissions was significantly higher in the total pandemic period (*p* < 0.001), and in all four virus predominance periods (cf. Table 1; all *p* < 0.05).

#### Length of stay (for inpatient cases)

The length of stay was not significantly different between the total study periods or during different virus predominance periods compared with the corresponding control periods in the inpatient cohorts (all *p* > 0.05).

### Disease burden (for inpatient cases)

#### Disease burden indices

The average sum of secondary diagnoses was significantly higher during all study periods than during the control periods (all *p* < 0.001).

The ECI was lower in the omicron period than in the control period (*p* = 0.002; cf. Table 1). The case mix index, a measure of expected resource use, was lower in the total and alpha periods than in the respective control periods (*p* = 0.005 and *p* = 0.01, respectively).

Several proportions of individual coded diagnoses of the ECI were significantly lower during the total pandemic period than in the pre-pandemic period: uncomplicated hypertension (*p* < 0.001), complicated hypertensions (*p* < 0.001), complicated diabetes (*p* = 0.044), renal failure (*p* = 0.010), liver disease (*p* = 0.017), and obesity (*p* < 0.001). None of the proportions of individual diagnoses from the ECI were significantly higher during the total pandemic period compared to the control period.

#### Complications

The proportions of several complications were lower during various virus predominance periods than during the pre-pandemic periods (Table 2; all *p* < 0.05): intensive care unit admission during the wildtype period, acute kidney failure during total, alpha, and delta periods, cystitis during the wildtype and omicron periods. The proportion of exsiccosis was higher during the wildtype study period than in the control period (*p* < 0.05).

**Table 2:** Frequency and proportions of complications in inpatient admissions for delirium

Complication, *N* (proportion)		Total		Wildtype		Alpha		Delta		Omicron	
		value	*p*	value	*p*	value	*p*	value	*p*	value	*p*
*Case number*	*Study*	*5362*		*1840*		*602*		*1108*		*1812*	
	*Control*	2358*		2396*		706*		1240*		2341*	
Intensive care unit admission	Study	637 (12%)	0.158	**204 (11%)**	**0.044**	75 (12%)	0.876	128 (12%)	0.527	230 (13%)	0.284
	Control	319 (14%)		**325 (14%)**		93 (13%)		169 (14%)		319 (14%)	
Mechanical ventilation	Study	30 (0.6%)	0.433	11 (0.6%)	0.733	3 (0.5%)	0.149	7 (0.6%)	0.742	9 (0.5%)	0.514
	Control	17 (0.7%)		17 (0.7%)		9 (1.3%)		7 (0.6%)		17 (0.7%)	
In-hospital all-cause mortality	Study	232 (5.1%)	0.347	76 (4.9%)	0.563	25 (5.0%)	0.880	47 (4.9%)	0.548	84 (5.5%)	0.223
	Control	91 (4.6%)		91 (4.5%)		29 (4.8%)		45 (4.4%)		91 (4.6%)	
Acute kidney failure	Study	**335 (6.2%)**	**0.010**	131 (7.1%)	0.397	**30 (5.0%)**	**0.035**	**58 (5.2%)**	**0.007**	116 (6.4%)	0.065
	Control	**183 (7.8%)**		184 (7.7%)		**56 (7.9%)**		**103 (8.3%)**		180 (7.7%)	
Exsiccosis	Study	1504 (28%)	0.590	**563 (31%)**	**0.010**	167 (28%)	0.855	281 (25%)	0.224	493 (27%)	0.868
	Control	669 (28%)		**679 (28%)**		202 (29%)		350 (28%)		659 (28%)	
Cystitis	Study	161 (3.0%)	0.108	78 (4.2%)	0.407	**11 (1.8%)**	**0.003**	35 (3.2%)	0.902	**37 (2%)**	**0.002**
	Control	91 (3.9%)		92 (3.8%)		**36 (5.1%)**		45 (3.6%)		**90 (3.8%)**	
Pulmonary embolism	Study	26 (0.5%)	0.248	7 (0.4%)	0.621	4 (0.7%)	0.821	5 (0.5%)	0.218	10 (0.6%)	0.210
	Control	7 (0.3%)		7 (0.3%)		4 (0.6%)		2 (0.2%)		7 (0.3%)	
Pneumonia	Study	365 (6.8%)	0.951	119 (6.5%)	0.763	31 (5.1%)	0.085	80 (7.2%)	0.156	135 (7.5%)	0.326
	Control	159 (6.7%)		162 (6.8%)		53 (7.5%)		71 (5.2%)		156 (6.7%)	
Thrombosis	Study	31 (0.6%)	0.271	13 (0.7%)	0.144	4 (0.7%)	0.557	8 (0.7%)	0.305	6 (0.3%)	0.777
	Control	9 (0.4%)		9 (0.4%)		3 (0.4%)		5 (0.4%)		9 (0.4%)	

* The control group for the “total” period refers to the full-year 2019 reference period. Control groups for individual virus predominance periods are date-matched 2019 intervals and may overlap; case numbers are therefore not additive and may exceed the full-year 2019 reference count. Bold print: significant difference between study and control periods (*p* < 0.05)

The graph depicts the temporal development of patient admissions for delirium in hospital-based outpatient (purple) and inpatient (green) settings from January 2019 to December 2022. During the COVID-19 pandemic, inpatient admissions decreased significantly, whereas the number of outpatient admissions was generally lower, although it slightly increased during the pandemic relative to the pre-pandemic levels (cf. Figure 2).

## Discussion

The COVID-19 pandemic has caused major disruptions in healthcare utilization. We present a retrospective analysis of routine data from 86 hospitals collected in Germany during the COVID-19 pandemic compared to data collected in pre-pandemic times. In this sample, there was an average admission deficit of 20% for inpatient cases with delirium as the main discharge diagnosis. Moreover, the proportion of coded comorbidities and complications in the inpatient cohort was largely unchanged or lower during the pandemic compared to the pre-pandemic periods. Importantly, the present analysis focuses on admissions with delirium coded as the main discharge diagnosis and therefore reflects hospital admissions primarily driven by delirium rather than the overall incidence of delirium in hospital settings.

### Deficit in inpatient admission was not compensated for by hospital-based outpatient admissions

Inpatient admissions for delirium were significantly reduced during the pandemic than during pre-pandemic periods. German and international data show no consensus on whether the frequency of hospital cases of delirium patients has changed due to the pandemic. Overall, inpatient admissions for neurological and psychiatric disorders have decreased in Germany [23]. Admissions for some neuro-psychiatric conditions varied, with cases coded from various disease categories becoming more common and others becoming less common in hospitals. Specifically, two regional analyses from western Germany reported that inpatient admission rates for delirium due to dementia were essentially unaffected during the pandemic [24, 25]. International data show mixed results. A Canadian study reported increased inpatient admissions for delirium during the pandemic [26], whereas a French study reported a significant decrease [5]. Taken together, these findings indicate that changes in delirium-related hospital admissions during the pandemic were heterogeneous across settings, while the present analysis demonstrates a remarked reduction.

Moreover, hospital-based outpatient admissions increased significantly during the pandemic. However, the magnitude was not sufficient to offset the reduction in inpatient admissions. These hospital-based outpatient services typically involve planned, time-limited diagnostic or therapeutic encounters without overnight stay and are distinct from primary care or community-based outpatient services. Hospital-based day care clinics are designed primarily to support rehabilitation-oriented goals, such as improving autonomy and activities of daily living in patients with neurological or psychiatric disorders. They are commonly used after discharge from inpatient treatment. In the present dataset, however, for every 13 missed inpatient admissions, only one additional outpatient case was detected during the total study period. Therefore, there was not simply a redistribution of cases to day care clinics, as we previously reported in admissions for dementia during the pandemic [27]. Notably, delirium has an acute onset and a fluctuating nature that requires continuous monitoring. Therefore, these findings suggest that the hospital-based outpatient services captured in this study were structurally limited in their ability to substitute for inpatient delirium treatment.

Overall, our multicentre dataset confirms a clear reduction in inpatient admissions for delirium not compensated for by hospital-based outpatient admissions. This finding becomes even more important when considering the pandemic effects that likely increased the risk for delirium and the need for hospitalization for delirium. The highly prevalent viral disease COVID-19 itself is a risk factor for delirium [6–9]. Additionally, delirium treatment is often resource intensive and requires a specialized and multidisciplinary approach involving neurology, psychiatry, internal medicine, and specialized nurses. The healthcare facilities were already overburdened and might not have been able to provide adequate care to patients with delirium (or those at high risk). Further protective factors against delirium were also reduced because of the pandemic: staff shortages, strict hygiene measures, and, very importantly, reduced contact (between patients and family or staff) [28–30]. The healthcare facilities were already overburdened and might not have been able to provide adequate care to patients with delirium (or those at high risk). Possible reasons for the reduction in admissions based on our observed data are discussed in the following section.

### Fewer delirium cases: changes in care pathways and access

The pronounced early decline in inpatient admissions exceeding 50% at the beginning of the wildtype predominance period suggests a substantial effect of multiple factors: triage decisions, access restrictions, and avoidance behaviour early in the pandemic. Given the abrupt pattern and timing of this decline, it is unlikely to be explained solely by changes in the underlying population-level risk of delirium. Rather, it points to system-level effects affecting hospital admission pathways for patients admitted for delirium. During the total study period admission rates for delirium remained consistently below pre-pandemic levels. There are several factors that may explain the reduced number of inpatient admissions for delirium during the COVID-19 pandemic. Some factors very likely reduced the need for hospital admissions for delirium because of reduced risk factors for delirium. Specifically, the postponement of (elective) procedures – especially surgeries – reduced the rates of anaesthesia- or surgery-induced delirium [1]. Improved outpatient or home-based management for cognitive conditions may have prevented some hospital admissions and therefore reduced the risk of delirium [31]. Other arguments could point to increased referral thresholds or altered admission practices for certain patient groups. For instance, referring physicians might have applied more restrictive triage criteria and therefore prevented hospital admission for high-risk patients with cognitive vulnerability [12]. Furthermore, concerns among patients and caregivers regarding nosocomial COVID-19 infection or other hospitalization-related risks may have contributed to delayed or reduced hospital admission [32].

To better understand the observed changes in admission patterns, we examined indicators of morbidity, complications, and mortality. Several indicators of disease severity and resource use, including the Elixhauser comorbidity index, selected secondary diagnoses, complications, and the case mix index, were lower or unchanged during the pandemic, suggesting no overall increase in case severity among admitted patients. On the other hand, the average number of coded secondary diagnoses was higher in all study periods than in the control periods. This latter finding must be interpreted with caution and does not necessarily indicate increased clinically relevant morbidity. The number of secondary diagnoses is highly sensitive to documentation and coding practices, which were substantially affected during the COVID-19 pandemic. Novel diagnostic codes, especially Z codes related to infection control, screening and hygiene measures, were frequently coded independent of patients’ underlying disease burden [33]. Taken together, it remains elusive whether the higher number of coded secondary diagnoses during the pandemic represented an increase in morbidity. Additionally, all-cause in-hospital mortality was unchanged between the pandemic and the pre-pandemic periods. These findings may seem counterintuitive. One might expect that the most morbid patients – given the urgency of delirium as a medical emergency – would have sought hospital treatment rather than having been managed in outpatient settings [34], which often have limitations in delirium care. This effect is also opposite to that of the general neurological population we analysed earlier. We reported significantly increased all-cause in-hospital mortality and increased morbidity indicators compared with the pre-pandemic periods, although at the same time we observed lower proportions of certain chronic comorbidities like congestive heart failure [12]. In line with those data, the present dataset of delirium cases also revealed lower proportions of specific known risk factors for severe courses of COVID-19 [35] in the pandemic cohort than in the pre-pandemic cohort. These risk factors included hypertension, renal failure, diabetes, liver disease and obesity. These increased risk factors may have increased the threshold for inpatient admissions on the side of patients, caregivers and referring physicians. Another possible explanation for reduced morbidity is that more morbid patients could have been classified not as delirium (as the main diagnosis) but as something else, such as depression, viral infection, or pneumonia [36, 37]. We advise that future research addresses this question further and that clinicians should be aware of the possibility of missed delirium diagnosis in multimorbid patients, especially during public health crises.

Elective admissions were reduced during the study period compared to pre-pandemic times. This observation likely reflects postponement or cancellation of planned hospital evaluations or follow-up admissions for delirium-related symptoms during the pandemic, rather than a change in acute emergency presentations. Importantly, elective admissions in this context do not necessarily imply mild disease. Instead, they comprised planned evaluations or follow-up admissions for persistent or recurrent delirium symptoms or admissions for unclear cognitive disorders. A possible explanation for an elective mode of some admissions was that the admission was initiated by a nonspecific cognitive disorder that was, in retrospect, diagnosed and coded as delirium. Adding to the missing alternative of outpatient clinics, it remains unclear where delirium patients were treated when they would have previously been admitted via the elective mode. One possibility is that admission was delayed until it was urgent and unavoidable. This would be harmful for the course of the disease since delayed treatment worsens the clinical outcome [37]. Another possibility is that delirium treatment was performed outside of the hospital network. Literature on outpatient treatment for delirium is very sparse [38].

Taken together, these findings might be more consistent with altered admission pathways and reduced access to hospital-based delirium care during the pandemic than with a simple reduction in the need for admission.

### Length of stay for inpatients with delirium remained unchanged

Despite other changes in healthcare utilization patterns during the COVID-19 pandemic, our analysis revealed that the average length of stay for patients with delirium remained unchanged. This stability could suggest that, once admitted, patients with delirium required similar inpatient resources and management times as they did before the pandemic. This effect was probably due to the complex and acute nature of delirium, which typically requires intensive, multidisciplinary care that seemingly cannot be shortened without impacting patient outcomes. This finding supports the interpretation that the observed reduction in admissions was not accompanied by a change in the intensity or duration of inpatient delirium care among those who were admitted.

### Limitations and strengths

This study has limitations, primarily due to its retrospective design. First, it lacks detailed information on different types of outpatient services. This limited the ability to assess whether other specific outpatient approaches may have compensated for reduced inpatient admissions. In addition, primary care, community-based outpatient services, and care delivered in long-term care facilities or nursing homes were not captured. That was because these services operate under separate reimbursement and documentation systems in Germany. Consequently, potential shifts toward “care in place” during the pandemic could not be assessed. Second, we did not analyse specific pandemic-related factors, such as staff shortages, resource constraints, and decreased patient-provider interactions, that might have influenced delirium management and patient outcomes. Third, these diagnostic codes may not always accurately reflect clinical diagnoses, although they are standardized by German laws, widely used for administrative purposes and frequently audited by independent health care organs. Fourth, the data are limited to delirium being the main diagnosis of hospital stay, excluding nuanced information on the development of delirium during the hospital stay or as a secondary diagnosis. This study does not capture the total burden or incidence of delirium in hospital-based settings. By design, it focuses on admissions *for* delirium rather than admissions *with* delirium. As a result, the observed admission deficit and other changes apply to hospital-based care pathways for delirium-led admissions rather than changes in delirium incidence. Fifth, due to the aggregated nature of the dataset and the lack of regional identifiers, the findings cannot be generalized to individual hospitals or directly extrapolated to healthcare systems in other countries.

Despite these limitations, the study also has important strengths. It is based on a large multicentre dataset from 86 hospitals, providing real-world evidence on hospital utilization patterns in routine clinical practice [39]. In addition, the longitudinal design covering multiple phases of the COVID-19 pandemic allows a detailed assessment of changes in admission patterns over time.

## Conclusions

This retrospective study revealed a striking 20% decrease in inpatient admissions for delirium in Germany during the COVID-19 pandemic compared with the pre-pandemic control period. As this decline was not compensated by hospital-based outpatient services, the findings indicate potential gaps in access to hospital-based delirium care during the pandemic. Changes in admission patterns and disease burden indicators suggest altered thresholds or pathways for inpatient admissions for delirium during the pandemic. These results underscore the importance of ensuring resilient and safe hospital care structures for delirium management during public health crises. These data inform the process of exploring alternative structures for delirium care when inpatient capacity is constrained. Such real-world data are essential for optimizing care pathways and strengthening preparedness for future healthcare disruptions.

## Data Availability

The datasets generated and/or analysed during the current study are not publicly available due to patient confidentiality but are available from the corresponding author upon reasonable request.

## Abbreviations

CI: Confidence interval
COVID-19: Coronavirus disease 2019
DRG: Diagnosis-related group
ECI: Elixhauser comorbidity index
GLMMs: Generalized linear mixed models
ICD-10: International statistical classification of diseases and related health problems, 10th revision
IRR: Incidence rate ratio
LOESS: Locally estimated scatterplot smoothing
OPS: Operation and procedure codes
